# Exploring the Optical and Morphological Properties of Ag and Ag/TiO_2_ Nanocomposites Grown by Supersonic Cluster Beam Deposition

**DOI:** 10.3390/nano7120442

**Published:** 2017-12-13

**Authors:** Emanuele Cavaliere, Giulio Benetti, Margriet Van Bael, Naomi Winckelmans, Sara Bals, Luca Gavioli

**Affiliations:** 1 Interdisciplinary Laboratories for Advanced Materials Physics (i-LAMP) and Dipartimento di Matematica e Fisica, Università Cattolica del Sacro Cuore, Via Musei 41, 25121 Brescia, Italy; giulio.benetti@fys.kuleuven.be (G.B.); margriet.vanbael@kuleuven.be (M.V.B.); Luca.Gavioli@unicatt.it (L.G.); 2 Laboratory of Solid State Physics and Magnetism, Department of Physics and Astronomy, KU Leuven, Celestijnenlaan 200D, B-3001 Leuven, Belgium; 3 EMAT University of Antwerp, Groenenborgerlaan 171, B-2020 Antwerp, Belgium; Naomi.Winckelmans@uantwerpen.be (N.W.); Sara.Bals@uantwerpen.be (S.B.)

**Keywords:** spectroscopic ellipsometry, metal and metal/oxide nanoparticles, supersonic cluster beam deposition, porosity, AFM, HAADF-STEM

## Abstract

Nanocomposite systems and nanoparticle (NP) films are crucial for many applications and research fields. The structure-properties correlation raises complex questions due to the collective structure of these systems, often granular and porous, a crucial factor impacting their effectiveness and performance. In this framework, we investigate the optical and morphological properties of Ag nanoparticles (NPs) films and of Ag NPs/TiO_2_ porous matrix films, one-step grown by supersonic cluster beam deposition. Morphology and structure of the Ag NPs film and of the Ag/TiO_2_ (Ag/Ti 50-50) nanocomposite are related to the optical properties of the film employing spectroscopic ellipsometry (SE). We employ a simple Bruggeman effective medium approximation model, corrected by finite size effects of the nano-objects in the film structure to gather information on the structure and morphology of the nanocomposites, in particular porosity and average NPs size for the Ag/TiO_2_ NP film. Our results suggest that SE is a simple, quick and effective method to measure porosity of nanoscale films and systems, where standard methods for measuring pore sizes might not be applicable.

## 1. Introduction

Coatings based on metallic nanoparticles (NPs) films, NPs dispersed in host dielectric or conductive matrices or barrier layers and hierarchical three-dimensional nanomaterials, are attracting a lot of interest due to their applicability in fields spanning from electro-catalysis [1], fuel cells [2], to antimicrobial coatings [3,4,5,6] and nanojoining [7,8]. However, to tailor the properties of nanostructured systems, the synthesis of nanoparticle-matrix nanocomposite coatings may require complex multi-step approach, even after the NPs synthesis, where matrix embedding may require colloidal stabilization, cross-linking growth and substrate functionalization [9]. 

A key factor in nanocomposite systems is the porosity [10,11], to obtain a large surface to volume ratio for increased catalytic performances or to tailor optical properties like plasmon resonances and near-field complex effects at the metal NP-metal NP or metal NP-matrix interface [12,13]. However, porosity is also a drawback with respect to the mechanical behavior, comprising critical technological issues like adhesion and wearing resistance. Porosity may be evaluated employing the Horvath-Kawazoe model [14], or the standard Brunauer–Emmett–Teller (BET) [15] and Barrett–Joyner–Halenda [16] methods, requiring reliable experimental isotherms and proper calculations [17] and are typically difficult to apply to ultrathin films. Porosity may be also deduced from mechanical indentation techniques [18] through a reduced effective elastic modulus of the film in comparison with the bulk materials. Unfortunately, the uncertainty on the Poisson modulus, related to the granular and porous nature of these layers, is too large and reduced elastic modulus may be not meaningful at all: moreover, nanocomposites have not a bulk counterpart concerning density and elastic properties and finally this method may irreversibly damage the film, at least locally. The non-destructive opto-acoustic method based on fs laser pump- and probe technique that we have recently employed to characterize elastic modulus, porosity and adhesion properties of Ag NP porous film [19] is very reliable but quite cumbersome and more applicable as benchmark method. 

In this work, the porosity and NPs size is deduced from a spectroscopic ellipsometry (SE) and morphological study of Ag NPs films and of Ag NPs embedded in porous titanium dioxide (TiO_2_) films, obtained in one step growth by supersonic cluster beam deposition (SCBD). The SCBD gas-phase synthesis method [20,21] allows to grow porous NPs films either metallic [6] or semiconducting [22,23] or even metal NP-dielectric nanocomposite films [24] directly from a suitable target source. A table-top spectroscopic ellipsometer (Woollam Alpha-SE) instrument allows indirect assessment of the film structure, especially the film porosity, through the information provided by optical properties of the film in the visible-infrared (IR) wavelength range and the use of a quite simple model.

## 2. Results and Discussion

### 2.1. Morphology and Structure of the Ag and Ag/TiO_2_ (Ag/Ti 50-50) Films 

**Figure 1:**
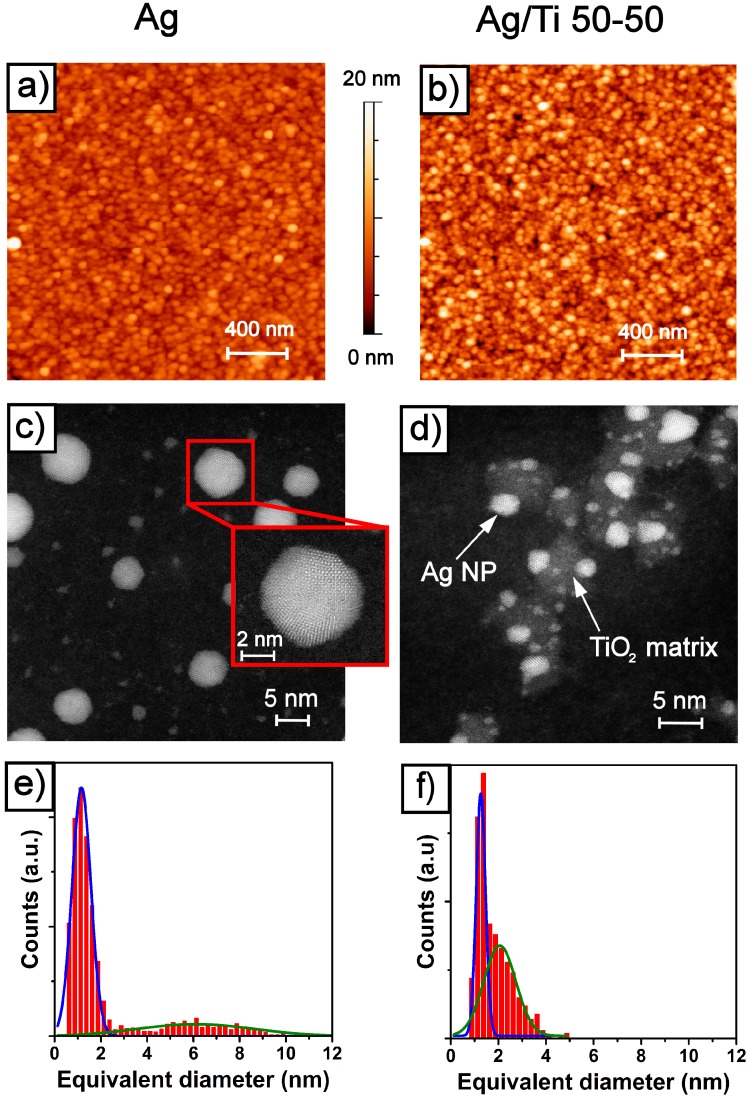
(**a**) AFM morphology of Ag NP film; (**b**) AFM morphology of Ag/TiO_2_ nanocomposite (Ag/Ti 50-50) film; (**c**) HAADF-STEM of low coverage Ag NP film deposited on Quantifoil TEM grid with ultrathin (5 nm thick) suspended C film; (**d**) HAADF-STEM of low coverage Ag/Ti 50-50 NP film on the same type of substrate; (**e**) Ag NP size distribution as extracted from a collection of 100 frames 50 × 50 nm^2^ wide ; (**f**) Ag NP size distribution as extracted from a collection of 70 frames 40 × 40 nm^2^ wide, only sizes of Ag NPs embedded in oxide matrix are considered. In frames (**e**,**f**) Gaussian fits are superimposed on the histograms to outline the bimodal size distributions of the Ag NP sizes.

Figure 1 outlines a prospect of morphology, structure and granulometry of the pure Ag NP film versus the Ag/TiO_2_ (AgTi 50-50) composite NPs films. The atomic force microscopy (AFM) data of Figure 1a,b, respectively, show that the 80 nm thick film of Ag NP and the 40 nm thick film of Ag/TiO_2_ 50-50 have quite similar surface morphology and a proper insight into the structural and chemical differences of the two films requires further investigations. The apparent lateral size of the NPs spans in the range 15–35 nm but, as already pointed out [6,19,24], actual lateral size of the NP in the film has to be estimated after deconvolution with the actual AFM tip apex radius (typical 10 nm) and by use of vertical height of isolated NP [25]. However, the root mean square (RMS) roughness of the two films are consistent with previous works: 1.7 nm for Ag film, 2.8 nm for Ag/Ti 50-50 nanocomposite. 

To yield details about the structure and grain size distribution of the films, a tiny fraction of monolayer (<0.05 ML, where a single monolayer is defined as 1 ML) of both bare Ag and Ag/Ti 50-50 composite were deposited on electron microscopy grids with amorphous carbon film for High Angle Annular Dark Field Scanning Transmission Electron Microscopy (HAADF-STEM). Figure 1c shows that pure Ag NPs are composed by different kind of nanocrystals. Larger NPs (5–10 nm diameter) present a polycrystalline twinned structure of fcc Ag, as evidenced in the inset of Figure 1c. The structure of nanocrystals smaller than 2 nm is more uncertain. Due to the lower melting temperatures of nano-crystalline than bulk Ag [26,27], the high power density of the incident HAADF-STEM beam is likely inducing a melting of such small NPs. Figure 1e shows the histogram of lateral size distribution of Ag NP, extrapolated from 100 frames acquired with the same scanning parameters adopted in Figure 1c. The size distribution is fit by a couple of Gaussian distributions: a narrower one for small objects population centered at 1.15 nm and with a 0.9 nm width and a broader one centered at 6 nm with a 5 nm width for the larger, twinned Ag nanocrystals.

The Ag/TiO_2_ nanocomposite (Ag/Ti 50-50) film shows a quite different structure, as shown in Figure 1d. Due to the atomic number sensitivity of the HAADF-STEM technique (the higher Z, the higher apparent brightness), Ag (Z_Ag_ = 47) nanocrystals are the brightest objects. The grayscale areas have been shown to be composed by a TiO_2_ matrix [24] related to the lower Z numbers of the components (Z_Ti_ = 22, Z_O_ = 8). The Ag NPs are partially encapsulated, more similar to the Janus-like than the core-shell configuration [28], the former being commonly observed for bimetallic noble metal nanoparticles [29]. Previous investigations about the structure of the oxide matrix performed by X-ray diffraction [24] suggested that the TiO_2_ is amorphous, in agreement with the TEM image shown in Figure 1d, where no sign of regular atomic arrangement can be observed in the “gray” areas.

In Figure 1f the size distribution of the Ag NP embedded in the amorphous titanium oxide, collected form a series of 70 frames, is reported. Even if the distribution is still bimodal as for the pure Ag case, with the smaller NPs population peaked around 1.2 nm, in the Ag/TiO_2_ nanocomposite the growth of larger Ag nanocrystal is hindered, since the data show a second peak at average diameter of 2 nm. This indicates that the presence of the amorphous TiO_2_ affects the size distribution of Ag NPs. Since the amount of TiO_2_ is directly related to the density of Ti in the starting rod for the ablation [24], this effect could be exploited to tune the size of Ag NPs. Moreover, modification of the Ti–Ag precursor rod with Mg–Ag, Al–Ag could lead to the formation of other Janus-type NPs or even multi element alloy NPs.

The film characterization performed by TEM yield information about the structure of the single components and about their size distribution, while the AFM data provides only a limited knowledge on surface roughness and thickness of the nanostructured film. Relevant information on the whole film, like porosity, mechanical properties and adhesion to the substrate remain to be explored.

### 2.2. Optical Properties by Spectroscopic Ellipsometry (SE)

The outputs of SE experiments are the dummy angles Ψ(*λ*) and Δ(*λ*) describing the function *ρ* (1), the ratio of the *p*-polarized and *s*-polarized complex Fresnel reflection coefficients *r_p_* and *r_s_*, respectively.

(1) $$\rho =\frac{r_{p}}{r_{s}}=\tan\left(\Psi \right)\cdot e^{i∆}$$

Hence the experimental Ψ(*λ*) and Δ(*λ*) data need to be modelled in order to obtain the physical properties of the film (see [30,31] for detailed treatment of the SE technique and principles). A proper model of the film is built from a stack of layers with their own associated dielectric functions, matching the properties of the film-substrate system. A very important factor that need to be taken into account is that the NP films discussed here are intrinsically porous, so a crucial void factor has to be introduced into the model.

**Figure 2:**
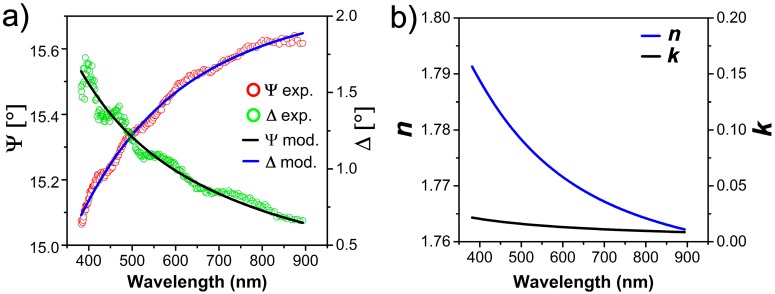
(**a**) Experimental data and fit of the Ψ and Δ angles for the Al_2_O_3_ substrate; (**b**) Cauchy-type model of infinitely thick Al_2_O_3_ substrate. Model *n* and *k* are shown.

As a reference, we also measured the bare Al_2_O_3_ substrate. The optical constants n and k of the bare Al_2_O_3_ substrate are modeled with an infinitely thick Cauchy-type layer available in the material library of CompleteEASE software and kept fixed throughout the simulations: experimental data with superimposed model and the optical constants of the Al_2_O_3_ substrates are shown in Figure 2a,b respectively.

**Figure 3:**
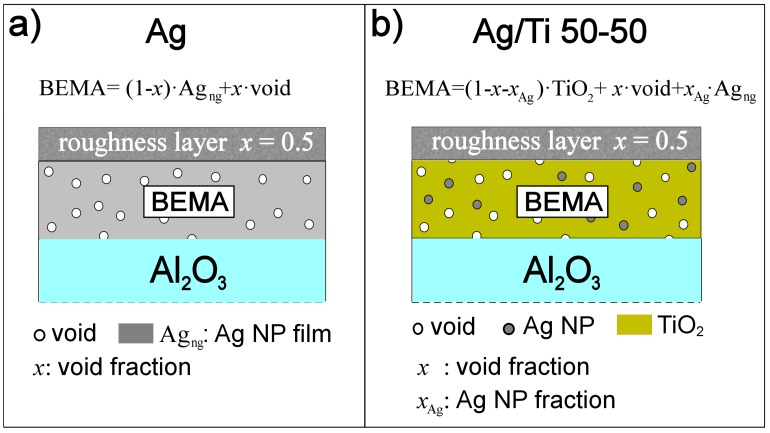
(**a**) layer model scheme for Ag NP film on Al_2_O_3_ substrate. The porosity of the film for both Ag and Ag/Ti 50-50 films is considered through the void factor (*x*) of the Bruggeman EMA layer (BEMA). Nano-granular Ag (Ag_ng_) is the host material; (**b**) layer model scheme for Ag NP/TiO_2_ (Ag/Ti 50-50) film on Al_2_O_3_ substrate. Amorphous TiO_2_ host material encapsulates a fraction of separated Ag NPs (*x*_Ag_) and voids (*x*). Surface roughness and possible gradients in the film void factor are considered through a roughness layer with fixed void factor *x* = 0.5.

The system is therefore modelled as follows: the NP films lies over Al_2_O_3_ substrate (Ag_ng_ in Figure 3a) and is represented with a uniform layer, considering also a void factor (*x* in Figure 3a) to represent the porous morphology.

For Ag NP films, any kind of chemical interaction or interdiffusion between sapphire substrate and the film is not expected, so we exclude any intermixing of the film-substrate layers. 

The porosity of the SCBD-deposited films (both Ag NP and Ag/Ti 50-50) is modeled within a Bruggeman effective medium approximation (BEMA) layer [32]: in Figure 3a continuous bulk Ag layer is the host material and void is the guest material, while in Figure 3b TiO_2_ is the host material and both Ag NPs and voids are guest materials. Depolarization factor is kept fixed to 1/3, so inclusions of guest materials will be considered as spherical.

A further topmost BEMA layer is introduced with a fixed void factor *x* = 0.5, to account for the surface roughness of the films (Figure 3).

The BEMA model could eventually fail to correctly reproduce dielectric constants of the film near the percolation threshold of the metal-void mixture. The analysis of X-ray reflectivity (XRR) oscillations on Ag NP films with the same thickness [19] estimated a void factor of 20%: so, it is quite likely that almost all NPs are in mutual electrical and mechanical contact and percolation threshold is comfortably exceeded. Parametrized bulk Ag optical constants are taken from [33] with a series of generalized oscillators available in the CompleteEASE software. The granular nature of the film is introduced with a correction Δ*ε* on the bulk dielectric functions *ε_b_* of silver metal [34,35], also known as FPE (free path effect) term due to size dependent surface plasmon resonance:

(2) $$\varepsilon =\varepsilon_{b}+∆\varepsilon =\varepsilon_{b}+\frac{\omega_{p}^{2}}{\omega }\left[\frac{1}{\omega +i\Gamma_{b}}-\frac{1}{\omega +i\Gamma \left(R\right)}\right];\;\Gamma \left(R\right)=\Gamma_{b}+A\frac{v_{F}}{R}$$

where the plasma frequency of bulk silver is *ħω_p_* = 9.2 eV and *Γ_b_* = 3.464 × 10^13^ s^−1^ is the scattering rate [36,37], *v_F_* = 1.4 × 10^6^ m/s [37] is the quasi-free electron velocity near the Fermi level and *A* is a variable parameter relative to the percolation of the NP film, spanning from *A* = 4/3 for dispersed nanoparticles, like in colloidal solutions [35] to *A* = 1 for continuous granular films [38]. *R* is the characteristic effective radius of the NP as probed by the electromagnetic radiation.

A similar approach was adopted by Bisio and coworkers to model SE data of Au NP films on oxidized silicon substrate, grown with a similar SCBD source. The model of bulk Au optical constants was corrected by the size dependent term Δ*ε*, similar to that appearing in Equation (2), to obtain the model dielectric function for the Au NPs films and even possible gradients of film densities and surface roughness were considered by implementation of multiple BEMA layers model with different void factors [39].

The layer thickness and roughness are determined according to the experimental RMS roughness values obtained from the AFM measurements (1.7 nm for Ag NPs film and 2.8 nm for Ag/TiO_2_ composites). 

The choice to model the film with oscillators layers ensures that the optical constant model is Kramers-Kronig consistent and so inherently physically meaningful, even if the matching between experiment and model maybe not perfect.

**Figure 4:**
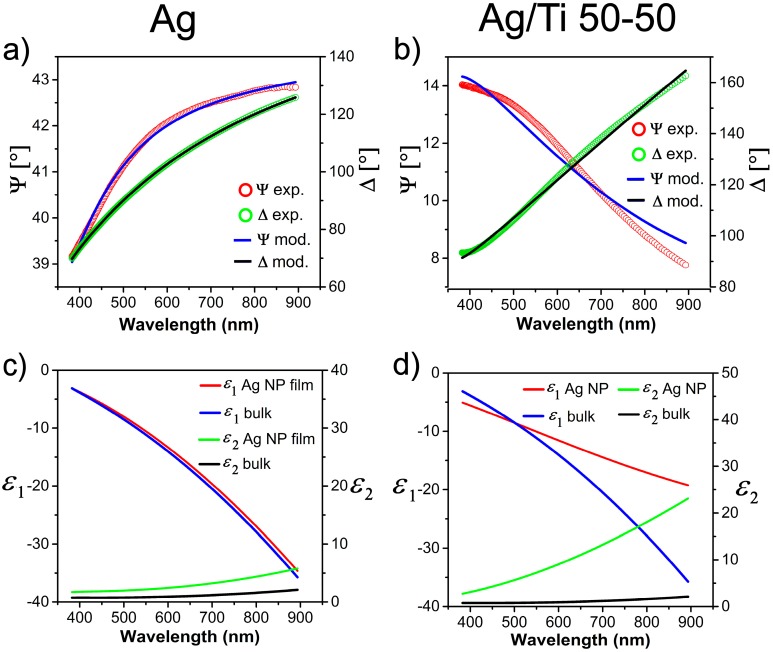
(**a**) Spectroscopic ellipsometry data and fit of Ag NP film with the model proposed in the main text; (**b**) Spectroscopic ellipsometry data and fit of Ag NP/TiO_2_ (Ag/Ti 50-50) film with the model proposed in the main text; (**c**) Plot of the model optical constants *ε*_1_ and *ε*_2_ of the bare Ag NP film compared with reference data [33] for bulk silver; (**d**) Plot of the model optical constants *ε*_1_ and *ε*_2_ of the Ag NPs in the TiO_2_ matrix for the Ag/Ti 50-50 film, compared with reference data [33] of bulk silver.

In Figure 4a,b the raw Ψ and Δ for the Ag NP and Ag/Ti 50-50 films are shown in comparison with the model results performed through the CompleteEASE software, based on the choice of oscillators discussed in the text and on the schematic model shown in Figure 3. The model dielectric constants *ε*(*λ*) *= ε*_1_(*λ*) + *i ε*_2_(*λ*) are shown in Figure 4c,d and compared with reference data used for bulk Ag [33]. The fit settings employed to model the Ψ and Δ experimental data are available in Appendix A (see Equations (A1) and (A2) in Appendix A).

**Figure A1:**
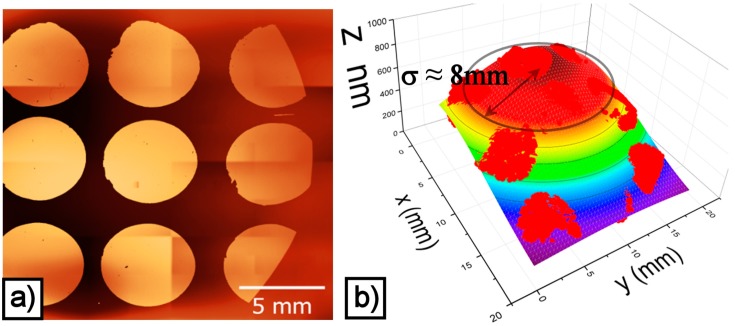
(**a**) 25 × 25 mm^2^ multi-tile optical profilometry image of a 700 nm thick Ag NP Film; (**b**) Optical profilometry data are fit with a bi-dimensional Gaussian profile with a parameter *σ* = 8 mm. The ellipsometer source light beam incident at 65° from sample normal projects a spot on the central region of the sample not larger than 4–5 mm: the maximum thickness gradient expected in the probed region of the sample is expected to be less than 5% of the maximum sample thickness. For an 80 nm thick layer expected thickness variation is less than 4 nm, very likely inside the expected surface roughness.

The model used for Ag NP film in Figure 4a looks quite effective in describing the optical properties of the medium. The film thickness from SE model is 87 ± 5 nm, in good match with the AFM measurements (80 nm). Also, the roughness layer thickness of 1.5 ± 0.2 nm variation is reasonably in tune with the actual film roughness (1.7 ± 0.2 nm) suggested by the AFM images. The void factor of the BEMA layer is *x* = 17 ± 1%, compatible the 20 ± 4% value from XRR measurements. Some uncertainty could come from thickness gradient in the film related to the SCBD beam shape. However, the data were taken in the film center, where the thickness gradient measured by AFM and profilometry changes are not more than 5% (see Figure A1 of Appendix A).

From the fit value of *ħΓ(R)* = 0.13 eV the extrapolated nanoparticle radius from Equation (2) is:

(3) $$R=\frac{\hbar \cdot A\cdot v_{F}}{\hbar \left(\Gamma \left(R\right)-\Gamma_{b}\right)}$$

so, if we set *A* = 1 in Equation (3), as suggested above, *R* ≈ 7.5 nm.

From TEM-deduced size distribution (Figure 1e), the average diameter of larger NP population is around 6 nm, while the extrapolated diameter or grain size from optical model (2*R* ≈ 15 nm) is more than twice this value. The picture of the optical constants of the Ag NP film devised by chosen oscillators could be not refined enough to fully explain the optical behavior of the film but actually it yields acceptable values for thickness and porosity. Since the value *R* deduced from Equations (2) and (3) is the radius of the NPs as probed by electromagnetic radiation, this suggests that the actual scattering length of the electrons is larger than the size of a single NP and influenced by the contact or coalescence of nearest neighbor NPs in the film. A tail in the distribution of larger Ag nanoclusters, reaching past 6 nm radius (12 nm diameter), is present but since it is so small it is likely to be not statistically relevant in the determination of the grain size.

**Figure A2:**
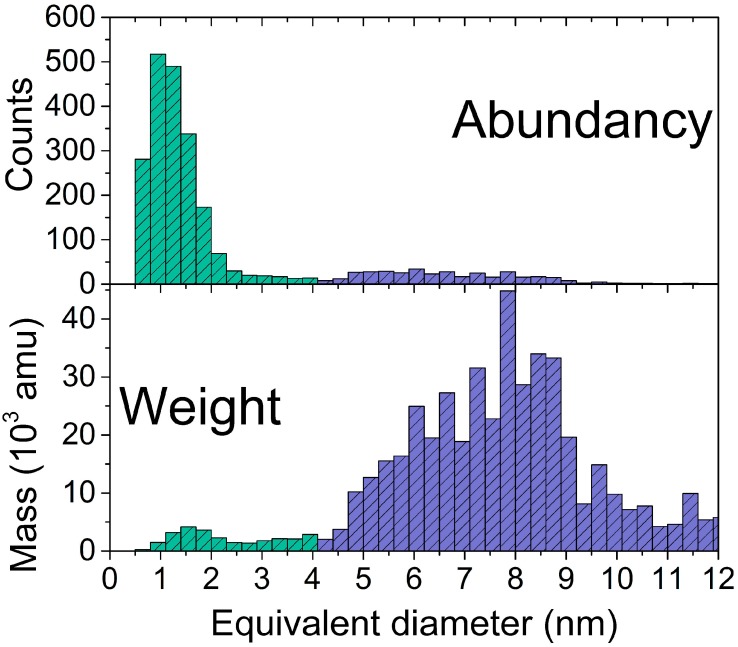
Size and mass distribution statistics of Ag NPs vs apparent diameters from TEM images. Particles with diameters less than 4 nm are identified in the green bins, while larger particles are collected in blue colored bins. Smaller than 4 nm NPs collect the 95% count of total particle number while their volume or mass is only 5% of total.

The population of smaller Ag NP (average radius 0.6 nm from Figure 1e), though it is around the 95% of the total particle count, looks like totally irrelevant in this model since the integrated mass or volume contribution of NPs with diameter smaller than 4 nm is only 5% of the total (see also Figure A2 in Appendix A). A possible explanation is also related to the fact that smaller nanoparticles (1.1 nm average diameter) in mechanical and electrical contact with much larger NPs (6 nm diameter) do not modify the effective size of the larger NPs. As shown by Bardotti and coworkers [40] and also in recent work by Benetti et al. [11], molecular dynamics simulations of these soft-landing films suggest that larger NPs also have lower specific energy (0.25 eV/atom) while smaller NPs land with specific energies around 1 eV/atom. These specific energies are not enough to produce structural modification by impact of a small cluster on a larger NP but they can induce coalescence or even epitaxial coordination of small nanoparticles impinging on larger nanoclusters. Finally, this phenomenon is not enough efficient to change the size of decorated, larger, effective NP. 

The model of Ag/Ti 50-50 film (Figure 3b) has to deal with the different nature of this film: the Ag NPs are basically embedded into the amorphous TiO_2_ [24], as shown by HAADF-STEM data of Figure 1d and such surrounding should result in totally non interacting Ag NP in the film.

The host TiO_2_ matrix is modelled with a Tauc-Lorentz oscillator, suited for amorphous materials [41], the guest materials in the BEMA layer approach will be Ag nanoparticles and voids, regarded as spherical inclusions in the host material, as shown in Figure 3b.

The oscillator terms due to optical interband transitions in bulk Ag will hence be neglected in favor of a simpler Drude model oscillator and only the correction for the size dependence of electron scattering term (FPE) is maintained, because is expected to be dominant for the typical size of our Ag NPs embedded (Figure 1d,f) and setting *A* = 4/3 in Equation (4).

(4) $$\varepsilon =\varepsilon_{\infty }+\frac{1}{\varepsilon_{0}\rho \left(\omega^{2}\tau +i\omega \right)}+\frac{\omega_{p}^{2}}{\omega }\left[\frac{1}{\omega +i\Gamma_{b}}-\frac{1}{\omega +i\Gamma \left(R\right)}\right];\;\Gamma \left(R\right)=\Gamma_{b}+A\frac{v_{F}}{R}$$

where, from [33], *ρ* = 6.7635 × 10^−6^ Ω∙cm and *τ* = 9.487 fs, $\varepsilon_{\infty }$ = 1.179. 

A similar model to describe the dielectric functions of silver NPs, accounting only for plasmon resonance and finite size effect in Ag NPs was early adopted by Kreibig [42]. 

The bulk-like terms need to be corrected by introducing the FPE term *Γ*(*R*), which accounts also for the effective reduction in the relaxation time *τ* in the Drude model when electron mean free path is comparable or larger than NP size [34,43]. 

This simplification is also suggested by the fact that at the TiO_2_/Ag interface electron transfer may happen when incident visible light excites electrons in confined Ag NPs at energies near or higher than the TiO_2_ bandgap (*E_g_* = 3.3 eV): this phenomenon was already observed in optical pump-and-probe experiments with infrared laser pump light probing confined Au nanodots separated by bulk Au substrate with a 4 nm thick TiO_2_ dielectric layer. The band-bending occurring at the metal-oxide interface partially reduces the energy barrier required for the excited hot electrons at Au nanodot/oxide interface to displace into the oxide barrier even with infrared light pump [44]. Our SE experiments were performed with visible light in the 380–900 nm wavelength range.

The average size of the Ag NP embedded in Titania matrix, from Figure 1f, is 2.5–3 nm. This leads to a typical Ag atom count in a NP to around 1000 atoms. Kreibig also reports in his work [42] that Ag NPs down to 400 atoms embedded in glass show usual metallic behavior. The energy spacing of possible discrete electronic levels due to electron confinement in the clusters should be lower than ~10^−2^ eV, quite similar to the energy provided by thermal excitation at room temperature. While hot electrons may be generated in the smaller (1 nm) particles [34], where levels maybe discrete, the thermal broadening of discrete levels in nanoclusters may smear effectively this discretization, resulting in a quasi-continuum of electronic levels. Hence it seems reasonable to assume the bulk Ag resistivity for the NP.

Since the NPs sizes in both Ag and Ag/TiO_2_ nanocomposites are smaller than the probing light wavelength, as shown in Figure 1e,f respectively, one could expect increased optical absorption of the medium due to the surface plasmon polariton (SPP) resonance in the Ag NPs embedded in the films [45]. This phenomenon should be even enhanced for insulated nanoparticles enclosed in a dielectric medium (as in Ag/TiO_2_ nanocomposites) as seen in reference [44]. The higher optical absorption than the one characteristic of bulk metals is also found in the model *ε*_2_ deduced for both of our films (Figure 4c,d) and coherently the metal NP-dielectric nanocomposite will show higher *ε*_2_ than nanogranular metal alone. 

It is interesting to make a comparison with a similar system to our Ag/TiO_2_ nanocomposites, an SCBD grown film of bare TiO_2_. Films of nanogranular amorphous TiO_2_ in the 50–100 nm thickness range show a 16–20 nm RMS surface roughness [46] while Ag/TiO_2_ nanocomposite film, 40 nm thick, (Ag/Ti 50-50) has only 3 nm RMS surface roughness. By SE measurements Toccafondi and coworkers [47] evaluated on these bare TiO_2_ films a film porosity larger than 50%. That value could be considered as un upper extreme to be allowed in the optical model, the lower extreme being the 20% of pure Ag NPs films found from XRR.

The difference in surface roughness between bare TiO_2_ and our Ag/TiO_2_ nanocomposite suggests that porosity of the film should stay in the middle of the two extremes above mentioned.

Further boundaries in the optical model are inferred from the composition and mass ratio of the film by X-ray photoelectron spectroscopy (XPS) on the films and X-ray fluorescence (XRF) [24]. From XPS the abundances or mass fraction of Ag and Ti are respectively 40% and 60% and from XRF 50% and 50% respectively for Ag and Ti. We point out also that XPS has probing depth of only few nanometers and mass ratio quantification is affected by different mean free path of photoelectrons with different energies in the probed layer: the initial mass abundance of the bulk rod target of 50% Ag and 50% Ti is almost transferred to the film.

Considering the bulk densities of Ag and TiO_2_, respectively *ρ*_Ag_ = 10.5 g/cm^3^ [48] and *ρ*_TiO2_ = 3.9 g/cm^3^ [49] the minimum mass ratio α_min_ = *m*_Ag_/*m*_TiO2_ set from XPS is ~0.67, while α_max_ = 1 (50% Ag, 50% TiO_2_), therefore we can state in Equation (5) that:

(5) $$\alpha_{\min}<\frac{m_{\mathrm{Ag}}}{m_{{\mathrm{TiO}}_{2}}}=\;\frac{{}_{\rho_{\mathrm{Ag}}}\cdot x_{\mathrm{Ag}}}{{}_{\rho_{{\mathrm{TiO}}_{2}}}\cdot \left(1-x-x_{\mathrm{Ag}}\right)}<\;1$$

The limits set in (5) are equivalent to the constraints on the volumetric fractions *x*_Ag_ (for Ag) and *x* (for void):

(6) $$\frac{\alpha_{\min}\cdot \rho_{{\mathrm{TiO}}_{2}}}{\rho_{\mathrm{Ag}}+\alpha_{\min}\cdot \rho_{{\mathrm{TiO}}_{2}}}\left(1-x\right)<x_{\mathrm{Ag}}<\frac{\rho_{{\mathrm{TiO}}_{2}}}{\rho_{\mathrm{Ag}}+\rho_{{\mathrm{TiO}}_{2}}}\left(1-x\right)$$

**Figure 5:**
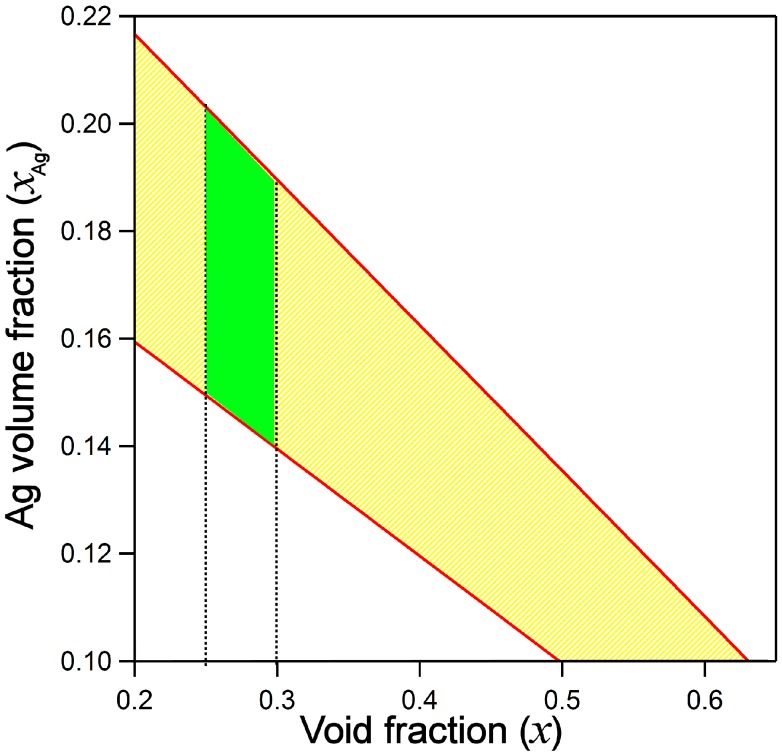
boundary values for void factor *x* (porosity) and *x*_Ag_ (volumetric fraction of Ag) are shown: allowed values lie in the yellow region of the graph, green area represents the value range for both *x* and *x*_Ag_ where fit of the ellipsometric data shows a minimum square error *MSE* < 10.

In Figure 5 the boundaries on volumetric fraction of Ag (*x*_Ag_) versus void factor *x* are reported and region of allowed values is highlighted in the yellow colored region. 

A meaningful model of SE data has to cope with topography data of AFM (40 nm height and surface roughness < 3 nm) and boundaries set by film chemical composition, as discussed above. Our model is more likely to give only a confidence band for the film porosity rather than a precise assessment: different trials were performed and best fit (mean square error MSE < 10) is obtained for *x* = 25 ± 5% porosity and volumetric silver fraction *x*_Ag_ = 18.5 ± 0.5%.

In Figure 4b we propose the best fit model for the Ag/Ti 50/50 nanocomposite film using Drude oscillator corrected with NP size effect (FPE) on dielectric function. The inferred thickness is 43 ± 1.7 nm and surface roughness 2 ± 0.4 nm, in good match with AFM measurements, *x*_Ag_ = 18.4 ± 0.7% and void factor *x* = 25 ± 5%, compatible with the limits set in Equation (6): these values fall into the green colored best fit region in Figure 5.

The superimposed optical model to SE data in Figure 4b only grasps the basic trend of the film response obtained from experimental Ψ and Δ data but there is a worse match with respect to the porous Ag NP film system.

Definitely, as shown in Figure 4d, it looks quite unlikely to match optical constants of the Ag NPs dispersed in the dielectric matrix only with corrections from bulk material, as done for bare Ag NP film in Figure 4c.

Our nanogranular Ag and Ag/TiO_2_ nanocomposite (Ag/Ti 50-50) films shows a typical surface roughness and porosity quite lower than the ones observed for other films deposited by SCBD, either the Au films analyzed by Bisio [39] or the bare TiO_2_ films in the works of Singh [46] and Toccafondi [47]. This difference could be traced back to different growth parameters of the sources, mainly the type of carrier gas (Ar or He) and different carrier injection pressure in the ablation chamber, anyway a sound motivation for these differences needs further investigation.

In our simple substrate-BEMA model layers we did not account for the effects at the amorphous TiO_2_/Al_2_O_3_ interface on the optical constants of the film. We choose on purpose a chemically stiff and inert sapphire substrate to avoid adding a further complexity into the modeling of these films. The interactions at the Ag NP film/sapphire interface will be mainly Van der Waals forces and weak interaction and adhesion is expected [50]. The deposition process in vacuum of this amorphous TiO_2_ matrix in Ag/Ti 50-50 films could leave chemically reactive defects like oxygen vacancies and dangling bonds persisting at the titania/sapphire interface, before they can be healed when the samples are removed from vacuum. These bonds could improve also the adhesion, due to the effect of stronger chemical rather than the Van der Waals bonds at the bare Ag NP/sapphire interface. A hint on this picture comes from the comparison of preliminary scratch tests performed on Ag NP and Ag/Ti 50-50 films, where adhesion of Ag/Ti 50-50 film on soda lime glass, probed by optical transmission after multiple scratch iterations, is way better than for bare Ag NP film [24].

A deeper knowledge of the interface with the substrate when the film is built with an amorphous oxide matrix is needed but even so the present SE measurements yield a consistent picture of the system predicting its porosity. This information is also very difficult to retrieve by XRR experiments, due to the large uncertainty on the effective density of the film.

Finally, the extrapolated effective radius of Ag NPs embedded in the host titania matrix from the *ħΓ(R)* = 1.0 ± 0.1 eV NP size-dependent scattering rate parameter (see Equation (3)) is *R* ≈ 1.2 nm, in good match with the average size of the Ag NPs population, as shown in Figure 1f.

## 3. Materials and Methods 

The principles of SCBD are described in detail elsewhere [6,22,25] and basically rely on the pulsed plasma ablation of a high purity metallic target rod, followed by NP condensation and expansion into vacuum through an aerodynamic lens/nozzle focusing system. A skimmer selects the central part of the beam directed on the substrate surface. In our experiments 99.99% purity Ag and Ag/Ti rods (ACI alloys, San Josè, CA, USA) were employed and the mass abundance of the elements in Ag–Ti bimetallic rod was 50% Ag and 50% Ti respectively. On both type of films, a mask was used during deposition in order to verify the film thickness by AFM measurement at film edges. A surface with both nanostructured film and bare substrate also allows comparison of the optical properties of the nanostructured films versus the bare substrate in the SE experiments. The nominal film thickness and deposition rate were measured by a quartz microbalance. 

Nanostructured Ag (Ag NPs) and Ag/TiO_2_ (Ag/Ti 50-50) films were grown at room temperature in medium vacuum conditions (base pressure 1 × 10^−6^ mbar) by SCBD directly on the substrate surface, in this case a 0.48 mm thick and 10 × 10 mm^2^ wide slab of (0001) α-Al_2_O_3_ single crystal, optically polished (MaTeck GmbH, Im Langenbroich, Juelich, Germany). The film deposited in vacuum were exposed to air to obtain titanium oxidation to TiO_2_ of the Ag/Ti 50-50 film.

Thickness, roughness and average grain of the films size were obtained ex-situ by AFM (Solver-P47pro from NT-MDT, (NT-MDT Spectrum Instruments, Zelenograd, Moscow, Russia), equipped with HA-NC “Etalon” tips from NT-MDT, with typical 140 kHz resonant frequency, 3.5 N/m force constant and tip radius of 10 nm, working in semi-contact mode.

Structure of scattered NPs, directly deposited on amorphous carbon grid (Quantifoil), was investigated by HAADF-STEM, performed with a probe corrected FEI TITAN microscope) and a FEI Tecnai Osiris microscope (instruments from Thermo Fisher Scientific, Hillsboro, OR, USA), both operated at 200 kV, respectively.

Optical properties of the film were obtained with a Woollam Alpha-SE spectroscopic ellipsometer (J.A. Woollam Co. Inc., Lincoln, NE, USA) in rotating compensator ellipsometer (RCE) configuration, working in the 380–900 nm wavelength range and the detector analyzes 180 points in this spectral range. The instrument is provided by the manufacturer with the CompleteEASE software tool, for data acquisition, modeling of film optical constants and integrated library of optical constants for a wide range of bulk materials and thin films. Experimental data were collected at different angles (65°, 70°and 75°) from the sample normal but for clarity only data acquired at 65° are discussed in the text. 

## 4. Conclusions

We investigated by spectroscopic ellipsometry the optical properties of Ag and Ag–Ti porous NP films grown in a single step by SCBD on Al_2_O_3_ substrates. The Ag films are composed of NPs with a bimodal size distribution, while the AgTi films are constituted by Ag NP surrounded by amorphous TiO_2_. The optical constants of the films are obtained employing a quite simple Bruggeman effective medium approximation model of the film structure. The model proves extremely useful to take into account the structure and morphology of the NP films, providing porosity and average grain size of Ag NPs in very good agreement with the ones obtained by complementary techniques. For the Ag/TiO_2_ NP film, the SE data provide porosity and grain size not obtainable by XRR or AFM and TEM, due to the large uncertainties about the effective density of the film itself. Moreover, our results suggest that SE is a viable method to obtain porosity measurements for nanoscale systems where standard BET method might not be applicable.

## Appendix A

In Equation (A1) we report the oscillators used to model Ag bulk material from [33] and implemented in CompleteEASE software interface:

(A1) $$\varepsilon_{B}=\;\varepsilon_{\infty }-\;\frac{1}{\varepsilon_{0}\rho \left(\omega^{2}\tau +i\omega \right)}+\overset{3}{\underset{k=1}{\sum }}A_{k}\frac{\Gamma_{k}}{2}\left(\frac{1}{\omega_{k}-\omega -i\frac{\Gamma_{k}}{2}}+\frac{1}{\omega_{k}-\omega +i\frac{\Gamma_{k}}{2}}\right)$$

$\varepsilon_{\infty }$ = 1.179
*ρ* = 6.7635 × 10^−6^ Ω∙cm;         Drude term
*τ* = 9.487 fs;

*A*_1_ = 1.206883 + *i* × 1.3885;    Interband oscillator harmonic terms
*ħ**Γ*_1_ = 6.8152 eV;
*ħ**ω*_1_ = 4.137 eV;

*A*_2_ = −0.674042 + *i* × 2.3861;
*ħ**Γ*_2_ = 0.3806 eV;
*ħ**ω*_2_ = 4.011 eV;

*A*_3_ = 4.525906 − *i* × 2.3944;
*ħ**Γ*_3_ = 22.148 eV;
*ħ**ω*_3_ = 2.723 eV;

Mean square error of the fit:
*MSE* = 1.445
Thickness = 87.2 ± 5 nm
Roughness = 1.5 ± 0.2 nm
*ħ**Γ*(*R*) = 0.13 ± 0.05 eV
*x* = 17 ± 1%

In Equation (A2) we report the oscillator used to model Ag NP optical constants inside the amorphous TiO_2_ matrix:

(A2) $$\varepsilon =\varepsilon_{\infty }+\frac{1}{\varepsilon_{0}\rho \left(\omega^{2}\tau +i\omega \right)}+\frac{\omega_{p}^{2}}{\omega }\left[\frac{1}{\omega +i\Gamma_{b}}-\frac{1}{\omega +i\Gamma \left(R\right)}\right];$$

$\varepsilon_{\infty }$= 1.179, from [33]
*ρ* = 6.7635 × 10^−6^ Ω∙cm;   Drude term from [33]
*τ* = 9.487 fs;

Mean square error of the fit:
*MSE* = 9.187
Thickness = 43 ± 2 nm
Roughness = 2.06 ± 4 nm
*ħ**Γ*(*R*) = 1.0 ± 0.1 eV
*x*_Ag_ = 18.4 ± 0.7%
*x* = 25 ± 5.2%        for BEMA layer

The mean square error fit goodness parameter (MSE) is defined as:

$$MSE=\sqrt{\frac{1}{3n-m}\overset{n}{\underset{i=1}{\sum }}\left[{\left(N_{i}^{exp.}-N_{i}^{mod.}\right)}^{2}+{\left(C_{i}^{exp.}-C_{i}^{mod.}\right)}^{2}+{\left(S_{i}^{exp.}-S_{i}^{mod.}\right)}^{2}\right]}\cdot 1000$$

where *n* is the number of considered wavelengths (180), *m* the number of fit parameters,

N=cos(2Ψ)
C=sin(2Ψ)cos(Δ)
S= sin(2Ψ)sin(Δ)
$N_{i}^{exp}$: *i*-th wavelength experimental *N* value
$N_{i}^{mod}$: *i*-th wavelength model/fit *N* value
$C_{i}^{exp}$: *i*-th wavelength experimental *C* value
$C_{i}^{mod}$: *i*-th wavelenght model/fit *C* value
$S_{i}^{exp}:\;$*i*-th wavelength experimental *S* value
$S_{i}^{mod\;}:\;$*i*-th wavelenght model/fit *S* value
